# Undifferentiated endometrial carcinoma diagnosed during perimenopausal hormone therapy: a case report and literature review

**DOI:** 10.3389/fonc.2024.1440246

**Published:** 2024-11-22

**Authors:** Ying Wang, Yu-Tong Zheng, Ling Zhang, Xue-Quan Cao, Zhu Lin, Hong-Yu Liu, Qiong-Ying Hu

**Affiliations:** The Taizhou Central Hospital (Taizhou University Hospital), School of Medicine, Taizhou University, Taizhou, Zhejiang, China

**Keywords:** UEC, perimenopause, hormone, curettage, ultrasound, MRI, pathology

## Abstract

Uterine corpus endometrial carcinoma, one of the three most frequent cancers of the female reproductive system, primarily affects women who are perimenopausal or postmenopausal. Moreover, it is an epithelial cancer that develops in the endometrium, which is classified as either estrogen-dependent (type I) or non-estrogen-dependent (type II). Non-estrogen-dependent endometrial cancers include plasma cell carcinoma and undifferentiated/dedifferentiated endometrial carcinoma. Undifferentiated endometrial carcinoma is a rare but aggressive endometrial epithelial cancer that lacks any histologic differentiation and is classified as a high-grade endometrial cancer. This case presents that a patient had uterine corpus endometrial carcinoma during perimenopausal hormone therapy, and the patient was treated with sequential estrogen–progestin treatment for 3 years. During that period, she did not undergo a follow-up examination for the last 2 years due to the pandemic. Undifferentiated endometrial carcinoma is a special type of endometrial cancer that is not hormone-dependent, and whether the occurrence of this case is related to perimenopausal hormone therapy needs to be verified by more evidence-based clinical cases and further studies.

## 1 Introduction

Uterine corpus endometrial carcinoma (UCEC), one of the three most frequent cancers of the female reproductive system, is an epithelial cancer that develops in the endometrium (1). It primarily affects women who are perimenopausal or postmenopausal (2). The morbidity of this condition makes it the sixth most common cancer among women worldwide, with a global age-standardized morbidity rate of 8.4/100,000 in 2022. Meanwhile, there were 97,723 cancer deaths from UCEC among women, and a global age-standardized mortality rate of 1.7/100,000 in 2022 (3). Additionally, an estimated 320,000 new cases are diagnosed annually (4). By contrast, the morbidity rate of UCEC in China was 6.8/100,000, accounting for 3.39% of the total morbidities of cancers among women, with a mortality rate of 1.1/100,000 in 2022 (5).The morbidity and mortality rates of UCEC have steadily increased, with a trend toward younger ages for the past 20 years (3, 6, 7), according to the data from the World Health Organization (WHO). The morbidity of UCEC has made it the most prevalent gynecological cancer in some developed countries, along with the decreased incidence of cervical cancer (8–12).

Based on pathogenesis and biological characteristics, UCEC is classified as either estrogen-dependent (type I) or non-estrogen-dependent (type II). The majority of estrogen-dependent UCEC cases are endometrioid adenocarcinoma, with mucinous adenocarcinoma making up a minority (13). Non-estrogen-dependent endometrial cancers include serous carcinoma, clear cell carcinoma, carcinosarcoma, mixed adenocarcinoma, undifferentiated endometrial carcinoma (UEC) or dedifferentiated endometrial carcinoma (DEC), mesonephric adenocarcinoma, squamous cell carcinoma, and gastric-type endocervical adenocarcinoma (14–16). As we know, UEC has only been reported a few times (17–20). UEC is rare and invasive and tends to be diagnosed at an advanced stage according to the International Federation of Gynecology and Obstetrics (FIGO) classification. Moreover, it is resistant to conventional chemotherapy (21, 22). Previously published papers have demonstrated a high risk of locoregional and distant recurrence in UEC compared with differentiated cancers (23); however, it has been published that the pathological and immunohistochemical features of these rare tumors received more attention. In addition, there is a lack of literature on their associated management and survival outcomes (24). This means that patients with UEC may face the dilemma of having to undergo only more invasive surgery.

Here, we presented a rare case of UEC that was diagnosed during perimenopausal hormone therapy. This is a rare case that was seldom reported before. At the same time, it is suggested that the long-term use of estrogen and progestin may influence the development of UEC. On the other hand, Dusp6 can be used as a biomarker to predict the effect of progesterone, and targeting MMR or SWI/SNF may become a trend in the development of UEC-targeted chemotherapeutics to provide a new strategy for the conservative treatment for UEC. The current report is expected to help us understand better the rare UEC and improve the treatment for UEC.

## 2 Case description

**Figure 1:**
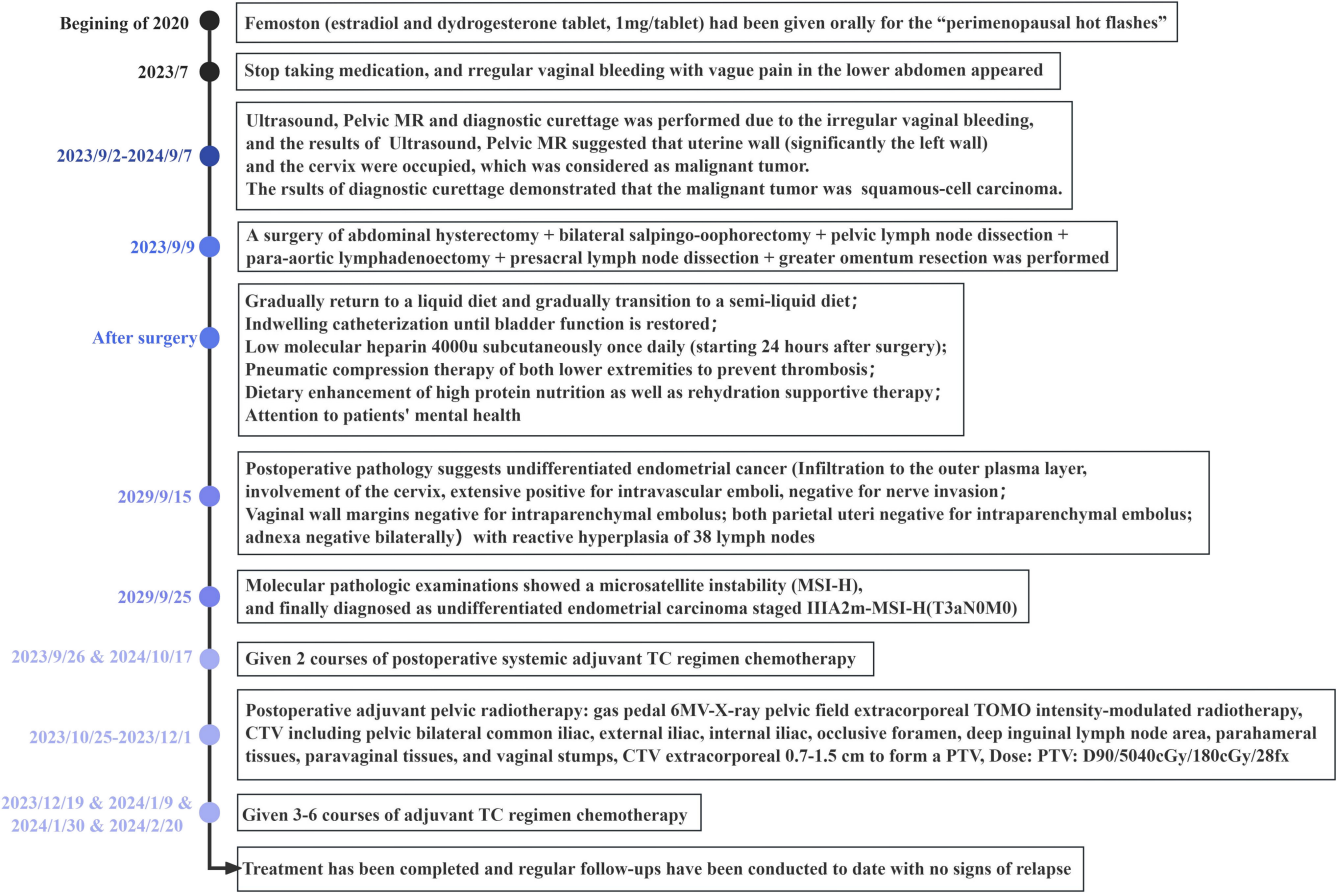
Clinical course timeline.

A 51-year-old female patient was admitted in September 2023 for experiencing “irregular and continuous vaginal bleeding for more than 2 months”. Her menstrual period stayed for 5~6 days, with a cycle of 28 days, and her last period was 30 August 2023. In addition, intermittent pain in the lower abdomen also occurred, but with no headache and dizziness, overflow of breast milk, difficulty in urinating, and/or other discomforts. Any additional treatment was not received during her period, but femoston (estradiol and dydrogesterone tablet, 1 mg/tablet) had been given orally 3 years before July for the reason of “perimenopausal hot flashes”. In addition, the patient did not have standardized follow-up examinations, because of inability to see a doctor due to the pandemic and personal situations. She underwent a “lower uterine cesarean section” in another hospital in 2000, accompanied by a spontaneous-labor, no-preterm birth. Moreover, any histories of genetic disease, mental illness, infectious disease, psychosocial history, or similar disease in the second or third generation of the family were denied. This was the first case of a patient who had UEC during the perimenopausal hormone therapy in this hospital (**Figure 1**).

**Figure 2:**
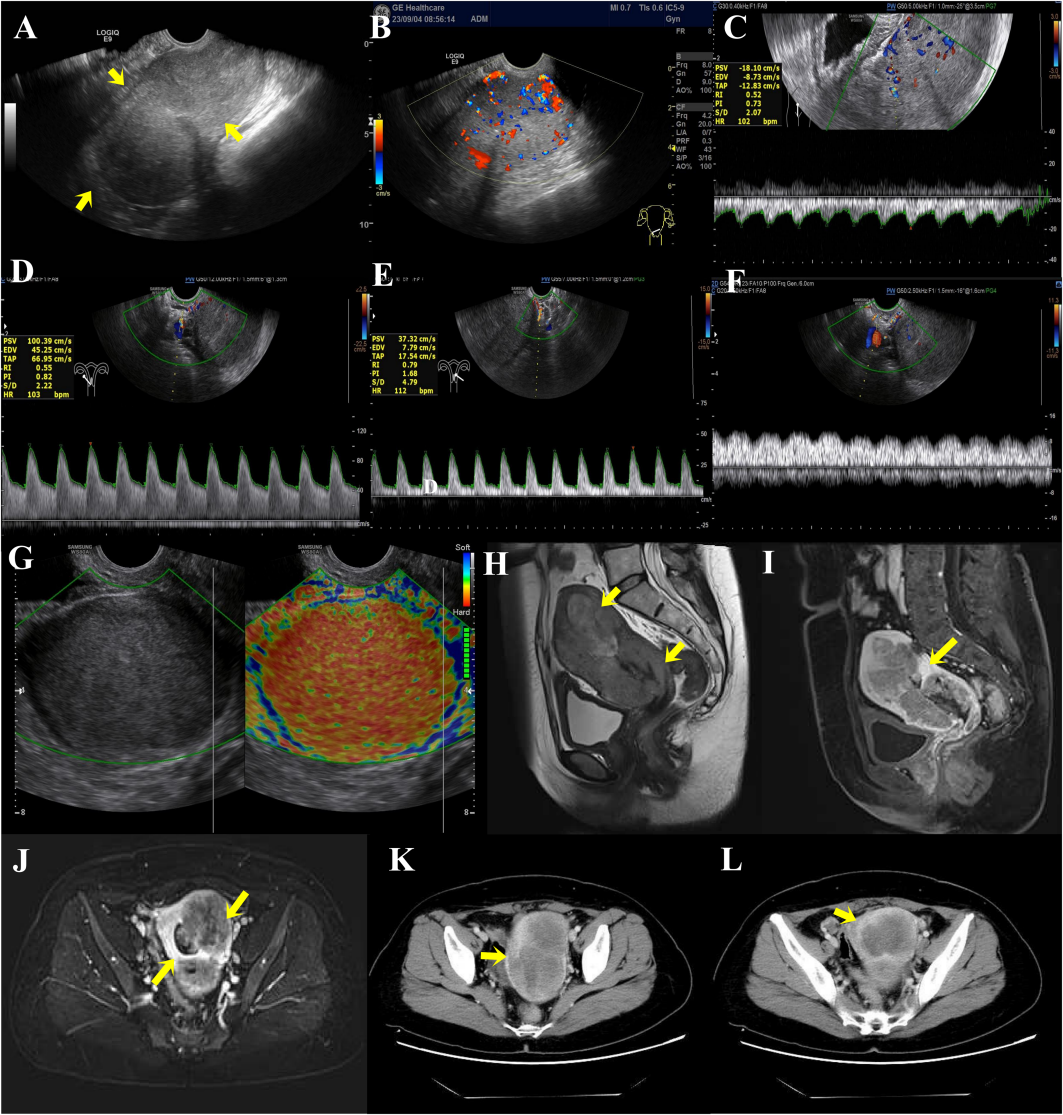
**(A)** Preoperative transvaginal color Doppler ultrasound sonogram of endometrial cancer lesions. Full uterine morphology, unclear cervical and uterine structures, and diffuse lesions in the myometrium (arrows show the location of infiltration of lesions). **(B–G)** Blood flow in and around the lesion. **(B)** Color Doppler ultrasound showed abundant blood flow signals in the interior and margins of the lesion. **(C)** Spectral Doppler ultrasound showed a low resistance type arterial flow spectrum detected within the lesion. RI: 0.52 (The general resistance index RI is less than 0.4, whereas the RI of this case is higher.) **(D)** The right uterine artery has increased blood flow and decreased vascular resistance. **(E)** Decreased vascular resistance of the left uterine artery. **(F)** Spectral Doppler ultrasound defined that the veins next to the uterus show a blood flow frequency spectrum like pulsation. **(G)** Transvaginal ultrasonography E-cervix elastosonography shows the lesion area in red, representing hard tissue. **(H–J)** MRI scanning and MRI enhancement. **(H)** Uneven abnormal signal mass in the cervix and left lateral wall of the uterus. **(I)** Uneven enhancement in the lesion of the myometrium and uterine cavity. **(J)** The local endometrial structures are poorly demonstrated, and the intrauterine lesion is poorly demarcated from the myometrial lesion. **(K, L)** CT plain scan and CT enhancement on the whole abdomen. **(K)** The uterus is enlarged in shape, with marked heterogeneous enhancement of the myometrium, predominantly in the left wall. **(L)** The lesion is poorly demarcated from the uterine cavity and endometrium.

Outpatient service was received. To diagnose the cause of the disease, we used ultrasound. The ultrasound showed a “long strip,” which was the echo of the intrauterine device in the uterine cavity, whereas the echo in the myometrium was not clear when the pressure was given, and that in the endometrium was not clear either (**Figure 2A**). Color Doppler flow imaging (CDFI) showed abundant blood flow signals in the area of the uterine cavity. The ovaries on both sides were slightly smaller than the average size, and the internal echogenicity was solid (**Figures 2B–G**). More details are shown in **Figures 2B–G**. The results of the spectral Doppler ultrasound presented a blood flow frequency spectrum like pulsation in the veins next to the uterus, which was possibly related to arteriovenous fistula.

Then, we used magnetic resonance imaging (MRI) to explore the abnormality in the uterine cavity. The MRI results of the pelvis showed an increased uterine volume, a thickened uterine wall (significant left side) and cervix, an irregular mass shadow and some small lymph node shadows next to the iliac vessels on both sides, along with approximately 10 mm in length for the large one, and uniform enhancement. In addition, the lesion invaded the endometrium and grew into the uterine cavity (**Figures 2H–J**). Analysis of computed tomography (CT) plain scan and CT enhancement on the whole abdomen showed the same phenomenon (**Figures 2K, L**). Given these findings, the mass may be classified as endometrial cancer.

**Figure 3:**
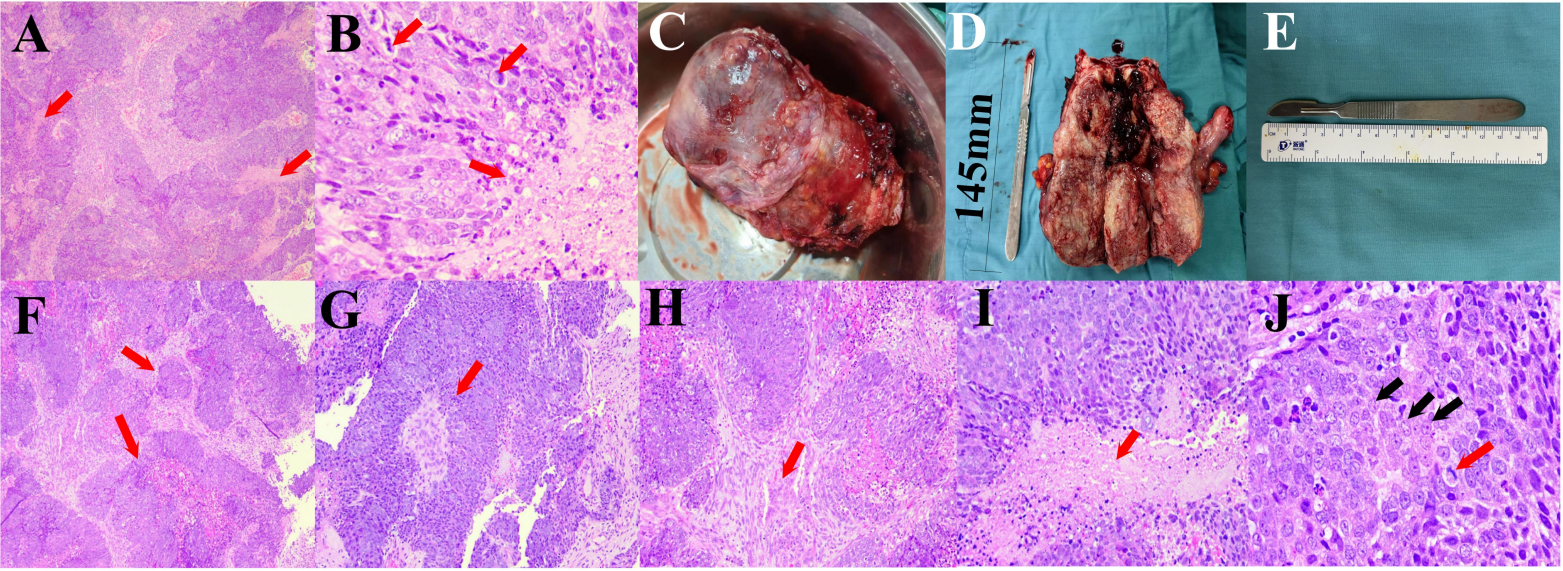
**(A, B)** Pathological results of diagnostic scraping. **(A)** The tumor has a nested structure with multiple coagulative necrosis (red arrows, 50×). **(B)** Coagulative necrosis is seen in the cancer nests (black arrows), the cells are round or polygonal, the nuclei are round and ovoid, the nucleoli are obvious, and nuclear division is common (red arrows, 400×). **(C–E)** The specimen of oophorectomy. **(C)** Enlarged uterus with lesion invading the surface of plasma. **(D)** The dissection specimen showed that the lesion invaded and grew into the entire uterus. **(E)** Length of the scalpel. **(F–J)** Pathological results of oophorectomy. **(F)** The tumor grows infiltratively, forming nested structures of varying sizes (red arrows, 50×). **(G)** Papillary structure with a fibrovascular core in the center (red arrow, 100×). **(H)** The cancer nests are of different sizes, and a desmoplastic response could be seen in the stroma (red arrows, 100×). **(I)** Coagulative necrosis is seen in the center of the cancer nest (red arrow, 200×. **(J)** Cells are large, round, or polygonal, with vacuolated nuclei and distinct nucleoli (black arrows), and nuclear division is common (red arrows, 400×).

To establish whether the uterine foreign body was indeed a malignant tumor, we performed a pathological examination after diagnostic scraping. The results of the pathology test suggest that the phenomenon of uterine cavity biopsy cells having much coagulative necrosis and a nuclear division is common, which is considered a malignant tumor (**Figures 3A, B**). Moreover, it was preliminarily considered as squamous cell carcinoma. Then, a surgery of abdominal hysterectomy + bilateral salpingo-oophorectomy + pelvic lymph node dissection + para-aortic lymphadenectomy + presacral lymph node dissection + greater omentum resection was performed. The adnexa of the whole uterus that underwent hysterectomy had the size of 13.0 cm*10.0 cm*5.5 cm and the tumor size was 11.5 cm*10.5 cm*5.0 cm. During the operation, some observations were obtained. First, the uterus was enlarged to a size greater than that of 2 months of pregnancy, and the cervix was thickened and hard, but the paracervical tissues were soft bilaterally. Second, scattered cauliflower-like tissues could be seen on the surface of the uterus. Third, there was a swollen lymph node in front of the sacrum and multiple swollen lymph nodes next to the abdominal aorta (**Figures 3C–E**).

After that, further pathological examination was performed to explore the type and stage of this endometrial cancer. The results of the immunohistochemistry test suggested that proteins P16, P63, CK5/6, CK-7, Vim, CK-20, and P40 showed positive expressions in minority of the lesion, CKpan and Ki-67 showed positive expressions in majority of the lesion, and WT1, P53 (wild-type), MLH1, MSH2, MSH6, PMS2, E-cad, and PAX-8 showed positive expressions. These suggested that the hysterectomy results showed cancer, specifically UEC (25). A molecular pathological examination showed microsatellite instability (MSI-H). Combined with the outcome of the postoperative pathological diagnosis and molecular pathological examination, it suggested that the stage of the UEC was IIIA2 m-MSI-H (T3aN0M0) (**Figures 3F–J**).

After surgery, the patient was treated with paclitaxel and carboplatin combined with systemic external beam radiation therapy. Right now, the patient is in good condition with the adjunctive treatment.

## 3 Discussion

During the course of perimenopausal hormone therapy for perimenopausal hot flashes for almost 3 years, the patient presented with typical symptoms of irregular vaginal bleeding for more than 2 months. The results of ultrasound, CT, and MRI showed that there was a foreign object in the uterine cavity and the endometrium became abnormally thick, which led to a preliminary diagnosis of endometrial cancer. Afterward, surgery was performed. Pathological analysis of the surgical specimen revealed that it was UEC, and it recovered well with subsequent paclitaxel/carboplatin adjuvant therapy. The rigorous adjuvant diagnostic measures and traditional diagnostic protocols in this diagnostic protocol led to a good prognosis for the patient. However, there were certain problems, for example, endometrial cancer types are difficult to differentiate, the surgery was more traumatic, the long-term use of paclitaxel and carboplatin may have led to the development of drug resistance in UEC, or the perimenopausal hormone therapy may have been linked to the emergence of UEC.

Based on this case, we summarize three experiences of diagnostic and management. (1) The first is medical imaging. If the uterine lesion has a wide range, invades the endometrium, and protrudes into the cavity to form mass-like changes with uneven enhancement, it will be initially considered as a malignant tumor of endometrial origin. That is exactly what happened in this case. Moreover, diagnostic imaging should focus more on the stage of the lesion, its relationship with neighboring tissues, the presence or absence of lymph node metastasis, distant metastasis, and other factors to provide detailed diagnostic reports to the clinic. As in this case, before surgery, comprehensive and systematic imaging examination was performed to determine the staging of the patient’s lesion, which is one of the reasons why the patient’s prognosis is favorable in this case. (2) Second is pathology. The morphology of undifferentiated endometrial carcinoma is similar to that of squamous cell carcinoma, serous carcinoma, or endometrioid adenocarcinoma, which is easy to be misdiagnosed. This is a problem that exists in all current endometrial cancer staging diagnostic procedures (15). However, further immunohistochemistry analysis can help in avoiding misdiagnosis and improving accuracy. Different treatment strategies exist for different pathological compartments of endometrial cancer (26); new biomarkers and well-defined pathological features can advance the diagnostic and therapeutic regime for undifferentiated endometrial cancer. (3) Third is clinics. There is a relationship between perimenopausal hormone therapy and the development of endometrial malignancy, which requires regular follow-up during hormone therapy. The patient was unable to see the doctor due to the pandemic, and therefore, she did not have standardized follow-up examinations, and this may have affected the progression of the disease.

Typical UEC is similar to lymphoma, high-grade endometrial stromal sarcoma, or small-cell carcinoma on morphology (27). The cells of UEC are almost uniform, medium-sized, poorly adhesive, and distributed in solid patches (23, 28–30). The molecular characteristics of UEC include inactivation of mismatch repair (MMR), the switch/sucrose nonfermentable chromatin remodeling (SWI/SNF) complex (28), and polymerase-epsilon exonuclease domain mutations (POLE-EDM) (31). Inactivation of the SWI/SNF complex is invasive in cases of UEC (28), but patients with POLE-EDM show a more favorable prognosis (31). It suggests that histologic diagnosis should be combined with molecular classification (32, 33). Excessive estrogen could induce UEC by inhibiting Succinate Dehydrogenase Complex Iron Sulfur Subunit B (SDHB) expression (34). Progestogens could increase the expression of progesterone receptor (PR), which is associated with abnormal regulation of MMR and the SWI/SNF (35–37). Recent studies have reported that the long-term use of progesterone may result in the absence of MMR (36) and that the transcription factor NF-E2-related factor 2 (Nrf2) is highly expressed during progestin treatment (38, 39). PR is also able to interact with hexanucleotide repeat expansion (HRE) exposed on the surface of nucleosomes and drive chromatin remodeling, which is a prerequisite for the activation of subsequent genes and determines the remodeling of nucleosomes by SWI/SNF (37). Additionally, it has also been reported that dual-specific phosphatase 6 (Dusp6) is useful for predicting progesterone response in endometrial hyperplasia, and its lack of expression significantly means the failure of potential treatment (40). It may serve as a biomarker for predicting response to progestin treatment. Due to the rarity, current treatment for UEC remains conventional, with practical experience limited to regular paclitaxel and carboplatin chemotherapy and/or radiotherapy (17, 41) and full-staged surgery (42). However, this case suggests that targeting MMR or SWI/SNF may be a strategy for developing targeted chemotherapy drugs for UEC in the presence of progestogens, providing a new approach for the conservative treatment of UEC.

## 4 Conclusion

Clear imaging features and pathological biomarkers help in diagnosis. In addition, it is necessary to measure the risks and benefits of surgery, radiotherapy, and chemotherapy for each patient. The case clarifies the impact of the prolonged exposure to estrogen and progestin on the development of UEC and suggests using Dusp6 as a biomarker to assess the effect of progesterone therapy. Moreover, targeting MMR or the SWI/SNF complex may emerge as a promising approach for developing conservative chemotherapeutic treatments for UEC.

## Data Availability

The original contributions presented in the study are included in the article/supplementary material. Further inquiries can be directed to the corresponding author.
